# Specialty Grand Challenge: Disability, Rehabilitation, and Inclusion

**DOI:** 10.3389/fresc.2020.622575

**Published:** 2021-03-04

**Authors:** Reuben Escorpizo, Thilo Kroll

**Affiliations:** ^1^Department of Rehabilitation and Movement Science, The University of Vermont, Burlington, VT, United States; ^2^UCD Centre for Education, Research and Innovation in Health Systems (UCD IRIS), School of Nursing, Midwifery and Health Systems, University College Dublin (UCD), Dublin, Ireland

**Keywords:** disability studies, rehabilitation, inclusion, patient and public involvement, international classification of functioning disability and health

## Disability: From Individual Model to System Challenge

Over the past century, there has been an evolution of thought about how “disability” is being viewed in cultural, sociopolitical, and legal terms (1). This had profound implications for education, research and practice in health sciences, and disability-related disciplines. Medical rehabilitation has traditionally regarded disability as a “defect,” a departure from “normal” physical or mental functioning resulting from congenital or acquired causes. Rehabilitation professionals would respond with interventions to mitigate the impact of impairments on people's lives. The tendency to view disability as a person's “problem” requiring treatment and correction has also been described as a medical or individual “model of disability” (2). This understanding has been challenged by disability rights activists since the 1960s who highlighted societal disenfranchisement and discrimination of people with disabilities. As part of the evolving understanding of disability as diversity, in the 1970s, the Centers for Independent Living were established to give voice and agency to people with disabilities (3). The Americans with Disabilities Act of 1990 was one of the first powerful legal instruments to challenge the social injustice and discrimination that people with disabilities experience in their daily lives. In 2006, the United Nations Convention on the Rights of Persons with Disabilities (UN CRPD) as a human rights legislation emphasized societal inclusion in all areas of life. Article 26 of the Convention specifically stresses the importance of habilitation and rehabilitation to support the goal of inclusion.

The shift from an “individual model” to a relational model of disability is also reflected in the WHO's International Classification of Functioning, Disability, and Health (4), a model that moved the rehabilitation community in strides toward a deeper understanding of inclusion. A relational understanding of disability refers to “disability” as the product of the interaction between the person and the environment. Consequently, efforts to make sure inclusion and rehabilitation have gone beyond “correcting” physical impairments of people. They now also address the social and environmental barriers that exclude people with disabilities from their full participation in the society. This effort also requires a multisectoral and interdisciplinary effort to be effective and meaningful. It also requires a critical eye for when and where people with disabilities are excluded, may it be in health care, research, in the community or society at large.

As an extension of the relational model, disability can be understood as a complex system challenge or “wicked problem” which transcends simplistic notions of individual impairment characteristics that manifest in homogeneous environments (5). Disability can arise based on situation, where environmental or social barriers exist, and it can become invisible in contexts that are accessible and inclusive of people with disabilities. People with disabilities live, learn, play, and work in different environments and they move between settings several times a day. Disability requires an ecological perspective and systems thinking to address its multifaceted consequences. People with disabilities are members of a group, may it be as part of a family, a student, an employee, as a friend, or as an athlete. They belong to different roles intersecting social systems which have their own dynamics. Rehabilitation professionals need to respond to a person's individuality and circumstances to tailor supports and interventions at the micro-, meso-, exo-, and macro-system levels as described in Bronfenbrenner's latest version of the ecological systems model, called Process–Person–Context–Time model (PPCT) (6). It is important to note that systems and environments can be characterized by objective features such as accessibility and availability of assistance but that they also carry subjective characteristics such as how friendly, welcoming, inclusive, or receptive environments are. A holistic view of rehabilitation and disability requires us to take into account the subjective as well as objective factors related to the person (e.g., demographic characteristics as well as psychosocial well-being) and the environment (e.g., built access barriers as well as environments that are perceived as non-receptive or not inclusive). We need to understand people in the context of the layered environments and system barriers and facilitators of inclusion- this approach brings in the value of rehabilitation into mainstream public health.

## Emergent Disabilities: Connecting Rehabilitation and Public Health

Further significant developments have occurred in public health. Traditionally, public health professionals focus on the prevention of “disabilities” resulting from preventable causes of illness and injury (e.g., accidents, illness, violence). Over the past 20 years, foremostly in the United States, public health professionals have recognized people with disabilities as a frequently disadvantaged population in relation to primary health care, health promotion programe, health risk behavior counseling, and public health campaigns and interventions (7). There is a clear need to close the gap between rehabilitation for acquired disabilities resulting from conditions like blindness, hearing loss, arthritis, depression, stroke, multiple sclerosis, spinal injuries, and the prevention of diseases such as diabetes, cardiovascular disease, cancer, HIV/AIDs through seamless collaboration among professionals across all sectors in the health care system.

The landscape of disability and rehabilitation is not static. New conditions with more or less complex impairments and rehabilitation requirements are continuously emerging. At present, we are only beginning to understand what the legacy of COVID 19 will be (8) and its long-lasting effect on the provision of rehabilitative care for people who experience disability and are already marginalized or stigmatized by the society. Rehabilitation professionals will have to be ready to respond to COVID's long-term consequences may it be neurocognitive or cardiorespiratory in nature. With this effort comes the technology to our disposal to help augment rehabilitation, while preventing disability and fostering inclusiveness.

## Rehabilitation and the Changing Technology Landscape

The last 30 years have seen unprecedented changes in the development of new technologies, especially in information and communication but also in relation to home, transportation and other aspects of daily living. People with disabilities are key users of technology in relation to mobility and navigation as well as in information-processing and communication. Increasingly, the boundaries between mainstream and assistive technologies are blurring (9). If this trend continues it may advance social participation and inclusion. Speech recognition technology is now widely used in business and in the communication with friends and family. Sensors and wearable devices (e.g., to detect falls, measure pulse, and heart rate etc.) have become mainstay technologies in mobile phones or smart watches. Ever more information is shared with unprecedented speed. New 5G technology will accelerate this process further. Big data linkage will give us novel insights into how people live, what they do and how they navigate their environments. Machine learning, artificial intelligence, and 3D printing will generate smart, adaptable systems, and devices (e.g., prostheses) that can be harnessed by health care professionals. Robotic systems will further revolutionize assistive capabilities in rehabilitation. The Internet has also opened up new ways for delivering rehabilitative interventions. Telerehabilitation may prove more feasible and effective than conventional rehabilitation approaches (10), which has been a primary model of healthcare delivery in the current pandemic. Patients may not be required to travel long distances for appointments. Conversely, exclusive or predominant reliance on technological solutions may inadvertently lead to further social marginalization of some. Not everybody will have equal access to these technologies. As a group, people with disabilities–while heavily reliant on assistive technology–earn less than the general population, are more likely to be underemployed and live-in poverty (11). Family members and personal assistants at times experience difficulty in accessing technologies that are useful for people with disabilities and also to stay current with updates. There is still a significant problem with the costs of assistive technologies compared to mainstream technologies (12). Affordability and education on how to use these technologies needs to be ensured. Otherwise, the goals of achieving equity and inclusion may be seriously jeopardized and disparity in technology access contribute to widening the gap in societal participation of people with disability.

The need for close involvement of stakeholders in the design of services, policies, or research is increasingly being recognized around the world (13). People with disabilities, therapists, personal assistants, and family caregivers have unique experiences, insights, expertise, and preferences that can ultimately lead to better technologies as well as enhance their acceptability (14). Timely and continuous service user involvement will also save money as products and services that are not fit for purpose or acceptable will be avoided and technologies will no longer be used and quickly abandoned. No matter how sophisticated, technology will fail if there is no cooperation among healthcare and rehabilitation providers. There is strong evidence to suggest that people from different disciplines working together to think of solutions to address disability is not only effective but is also cost-efficient. A multidisciplinary approach becomes more imperative as we continue to see increasing prevalence of multimorbidity.

## Toward People-Focuses Pathways: Recognizing Multimorbidity and the Need For Multi-Condition Rehabilitation Pathways

Rehabilitation is a complex process and involves multiple stakeholders. Patients or consumers who are being referred to rehabilitation require the optimization of their functioning and a meaningful return to their daily lives including work and employment, life activities, school, and community affairs. Co-existing or multiple morbidities can complicate and make negatively impact a person's activity and participation and thereby will challenge the rehabilitation process (15). Increase in comorbidities has a disproportionate high cost, high impact on quality of life, and well-being. It increases the demand for care coming from the person's families and caregivers. Multimorbidity has been defined as having two or more diseases or health conditions (16), with an overall prevalence of 33% among high, middle, and low-income countries (17). Multimorbidity poses a challenge in how rehabilitation outcomes are assessed because of its confounding effect on the outcome; one cannot fully take into account the effect of every single morbidity because of the possibly overlapping symptoms, varying stages of severity and recovery, and time of onset. One way to understand the effects of multimorbidity is to look at cases longitudinally so we can develop different pathways or trajectories that capture the predominant characteristics of an individual's functioning as it relates to their co-existing health conditions.

Moreover, rehabilitation and other health care professionals will be increasingly confronted with complex challenges arising from people with multiple health care conditions (multi-morbidity). Rehabilitation would not only have to take into consideration single (e.g., stroke, cardiovascular disease), but multiple conditions (e.g., stroke and arthritis; dementia, brain, and spinal injuries). A patient-centric rehabilitation approach is critical now more than ever given the landscape of changing and emergent needs in rehabilitation research and practice, and the resultant socioeconomic environment that may magnify the deleterious effect on inclusion of people with disability. Continuing to involve the users and the patients in rehabilitation practice and research will only benefit the dialogue between researchers and users, finding ways that are sensible to the end-users and robust from research and outcome measurement perspective. Ultimately, we want to achieve people-centered, not only condition-specific pathways of care.

## “Having A Say”: Public and Patient Involvement

Public and patient involvement is a cornerstone in disability research and practice. In partnership with patients/users, rehabilitation espouses collaboration between disciplines toward the aim of improving patient outcomes. The philosophy of “nothing about us without us” is fundamental. If we were to understand, assess, and help with the rehabilitation of people who may be excluded from the society because of their disability. Recent efforts in the rehabilitation field have focused on engaging patients or consumers in a way that they participate in the design and conduct of the research and acting as consultants (18), where patients are equal partners in research. When patients become active partners, research benefits from the information that otherwise would not have been known to the researchers. Patient research partners can provide insight into what works and what does not work from a practical perspective or when studies get implemented, such as in participatory or “community-grassroot initiatives.” Patients, as stakeholders, become engaged in the research not as passive study participants but rather as co-researchers. They become instrumental in the process of study development, conduct and dissemination.

Concrete efforts can be made and promoted in this field. For example, patients can participate as members of the research steering committee, or as members of an Advisory Board (19). Patients can also be members of decision-making bodies with funding research projects (18). This contemporary notion of having patients as active partners in research does not only rely on the direct involvement of patients in research but also points out to a crucial matter of providing common language and lay terminology of research products and findings that will enhance consumer education so people can have an informed decision. Truly, patient-centredness is key to rehabilitation research and practice. Research efforts has increased in work disability research due to its direct impact on societal productivity and the economy.

## Leveling the Playing Field: Rehabilitation, Employability and Income Inequality

Disability can impact different levels of functioning from the person to the family to the extended family to the community and the society. The nature of this impact requires that rehabilitation also address the different levels of functioning accordingly and appropriately. Recent models have come to light such as the Disability Evaluation, Livelihood, and Employment Rehabilitation Model (DELIVER) (20). The DELIVER model is a comprehensive look at work disability using multiple lenses of Individual worker capacity to provision of worker rehabilitation to the value of sound policy and economics. The current pandemic has brought about a real negative impact on employment rate and productivity secondary to economic shutdown amidst public health concern. This situation has led to workers with disability being at a disadvantage compared to the general working population. The state of the economy has led to rationing of resources and further marginalized workers with disabilities who are now struggling not only to provide means but also being able to find suitable jobs that will match their ability or capacity but recognizing the limited number of opportunities. Individualized assessment of the worker is essential in any discussion of return-to-work strategy (21), and would require a systems-level approach (22). Rehabilitation research and practice is crucial now especially given the COVID-19 pandemic because it brought to light the potential of worsening access to services desperately needed by people with disability and disproportionate “rationing” or re-prioritization of resources.

## Conclusion

Fragmentation and specialization in rehabilitation have distanced the disciplines from the diverse and complex lives of people with disabilities. Simple technological “fixes” will not be able to transform rehabilitative care to address emergent and complex rehabilitative care and support needs. A rehabilitation system of the future would have to return to the root of truly capturing the input of people and communities to identify the needs and influence effective practice of care. Patients, families, and communities are key stakeholders in rehabilitation and need to be recognized as co-designers of health research and services if we are to combat disability. If scientifically robust rehabilitation is co-created, implemented and evaluated in collaboration with diverse community stakeholders, we can create a holistic, context-sensitive approach to people's lived experience of their disability.

## Author Contributions

RE and TK equally contributed in the conceptualization, writing of the manuscript, and approved the manuscript prior to submission.

## Conflict of Interest

The authors declare that the research was conducted in the absence of any commercial or financial relationships that could be construed as a potential conflict of interest.
